# The role of *Desmodium intortum*, *Brachiaria* sp. and *Phaseolus vulgaris* in the management of fall armyworm *Spodoptera frugiperda* (J. E. Smith) in maize cropping systems in Africa

**DOI:** 10.1002/ps.6261

**Published:** 2021-01-28

**Authors:** Laetitia Scheidegger, Saliou Niassy, Charles Midega, Xavier Chiriboga, Nicolas Delabays, François Lefort, Roger Zürcher, Girma Hailu, Zeyaur Khan, Sevgan Subramanian

**Affiliations:** ^1^ Haute école du paysage, d'ingénierie et d'architecture de Genève Geneva Switzerland; ^2^ International Centre of Insect Physiology and Ecology Nairobi Kenya

**Keywords:** intercropping, push‐pull, legume, deterrent, Lepidoptera, maize

## Abstract

**BACKGROUND:**

The fall armyworm (FAW), *Spodoptera frugiperda* (J.E. Smith) is a serious pest of maize. Farming systems such as push‐pull or maize‐legume intercropping have been reported to reduce FAW infestations significantly. However, the exact mechanisms involved in FAW management have not been practically elucidated. We therefore assessed larval host preference, feeding and survival rate when exposed to four host plants commonly used in push‐pull and legume intercropping. We also compared adult moths' oviposition preference between maize and other grasses used as trap crops in push‐pull.

**RESULTS:**

The larval orientation and settlement study showed that maize was the most preferred host plant followed by bean, desmodium and *Brachiaria brizantha* cv Mulato II. The larval arrest and dispersal experiment showed that mean number of larvae was significantly higher on maize than on *Desmodium* or *B. brizantha* cv Mulato II. However, no significant differences were found between maize and bean after 24 h. Maize was the most consumed plant, followed by bean, desmodium and finally brachiaria. The mean percentage of survival to the pupation stage was significantly higher on maize. The study on FAW oviposition preference showed no significant differences in egg deposited between maize and other grasses. However, *B. brizantha* cv Xaraes, which received more eggs than maize, could be a promising alternative to *B. brizantha* cv Mulato II for the control of FAW.

**CONCLUSION:**

The study provides a better understanding of the mechanisms involved in the control of fall armyworm under the push‐pull and maize legume intercropping. © 2021 The Authors. *Pest Management Science* published by John Wiley & Sons Ltd on behalf of Society of Chemical Industry.

## Graphical abstract

Goal: Demonstrate how push‐pull and maize‐legume intercropping control fall armyworm. Major findings: FAW neonates prefer maize followed by beans. Desmodium is deterrent to FAW neonates. FAW neonate do not feed on brachiaria. No significant difference in egg laid on maize and various brachiaria. Outcome: Better understanding of agroecological management of FAW using push‐pull and legume intercropping. The need for adaptive research in the identification of attractive plants for FAW moths. Need for more research on the role of intercrops on below‐ground effect on crop health.

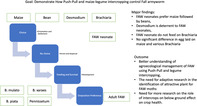

## 1 INTRODUCTION

Cereal crops play a vital role in the daily diets of Africans and account for up to 46% of total calorie consumption.^1^ Maize (*Zea mays* L.), for instance, is a major staple food crop cultivated in diverse agro‐ecologies in sub‐Saharan Africa (SSA).^1^ In 2017, global maize production added up to 1.04 billion tonnes, of which close to 15% was traded on international markets.^2^ However, maize production is constrained by key abiotic and biotic factors, which expose small‐scale farmers to food insecurity and vulnerability. Biotic constraints, such as stemborers *Chilo partellus* and *Busseola fusca*,^3^ the parasitic weed *Striga* spp.^4^ and diseases^5^ are aggravated by climate change stressors such as changing temperatures, extended droughts and unpredictable rainfall patterns.^6^, ^7^

The fall armyworm (FAW), *Spodoptera frugiperda* (J. E. Smith) (Lepidoptera: Noctuidae), a cereal pest native to the Americas that recently invaded Africa, overloads maize production with challenges.^8^ Losses attributable to FAW are huge; crops worth over $US2.4–6.2 billion per annum in sub‐Saharan Africa are at stake.^9^, ^10^ Consequently, the livelihoods of millions of poor farmers of cereal, forage and grass, as well as small‐ and commercial‐scale seed producers in SSA, are threatened.^10^, ^11^

Chemical insecticides are the primary control strategy against FAW; however, these are not affordable to smallholder farmers in Africa. Furthermore, the nocturnal habits of adult moths and the cryptic and burrowing behaviour of larvae into the maize whorl renders control difficult.^11^ In addition to that, FAW has developed resistance to many chemical insecticides.^12^, ^13^ Excessive use of chemical insecticides also raises health and environmental concerns.

Several agroecological management practices have been reported to effectively control FAW.^14^ For example, recent studies have demonstrated that push‐pull technology significantly reduces FAW infestations in maize.^15^, ^16^ Push‐pull is a ‘stimulo‐deterrent diversion’ tactic, whereby gravid stemborer females are repelled from the maize crop by the intercrop *Desmodium* spp. and are simultaneously attracted to a trap crop (*Brachiaria* spp. or Napier grass, *Pennisetum purpureum*). Napier grass and brachiaria produce significantly higher levels of attractive volatile compounds during the scotophase, which attract gravid stemborer females for oviposition. However, about 80% of stemborer larvae do not survive, since Napier grass and brachiaria are not suitable plants for their development.^17^, ^18^ The performance of the technology therefore depends on the degree of attractiveness and of repellence of the intercrop. Initially developed for stemborers, the strategy by which the technology controls the new invasive pest FAW has not been elucidated, although it can be assumed that the same control mechanisms as for stemborers, such as *Busseola fusca* or *Chilo partellus*, might prevail.^19^

Furthermore, intercropping with edible legumes, such as cowpea, groundnut soya and common bean, can also reduce FAW infestations considerably on maize.^16^ Indeed, FAW is a noctuid and feeds on more than 100 host plants belonging to several plant families, although maize is the most preferred host plant.^19^, ^20^ However, the direct effects of push‐pull companion plants and edible legumes on FAW host preference, feeding and fitness, and oviposition have not been established yet. Such information is crucial for the enhancement and resilience of agroecological maize farming systems, such as push‐pull and legume intercropping, against FAW.

This study aims to elucidate the effects of *Desmodium intortum*, *Brachiaria* spp. or Napier grass *Pennisetum purpureum* and bean used in push‐pull and maize‐legume intercropping on the behaviour of FAW.

## 2 MATERIALS AND METHODS

### 2.1 Study site

The present study was conducted at the International Centre of Insect Physiology and Ecology, Thomas Odhiambo Campus (ITOC), Mbita Point, situated on the eastern shores of Lake Victoria (0°260′06.1900′′S, 34°120′53.1300′′E, 1137 m above sea level) in western Kenya.^21^ The vegetation is of savannah grassland type, with mixed combretum and acacia trees to the north and papyrus along the shores of the lake. The average annual precipitation is 900 mm, with an average annual temperature of 27 °C. The mean temperature inside the laboratory was 25.5 °C during the day and 23.5 °C at night, with 70 ± 5% relative humidity (RH) and natural light conditions of approximately L12:D12.

### 2.2 Plants

The plant species selected for the experiments were directly procured on station. They included maize, *Zea mays* L.; Bean, *Phaseolus vulgaris* L. var. Roscoco; Greenleaf desmodium*, Desmodium intortum* (Mill) Urb. and *Brachiaria* cv. Mulato II (*B. ruziziensis* × *B. decumbens* × *B. brizantha*). Smallholder farmers in the region commonly use the maize and bean varieties selected in this study. *Desmodium intortum* and *Brachiaria* cv Mulato II are the companion plants used in climate‐smart push‐pull technology. All plants used in this study were grown in pots in screen houses at ITOC.

The two‐choice oviposition tests with FAW moths were conducted following a procedure adapted from Sokame et al. (2020).^22^ FAW oviposition preference was evaluated using the following trap plants: *Pennisetum purpureum*, *Brachiaria* cv Piata*, B*. cv Xaraes and *B*. cv Mulato II. *Pennisetum purpureum* and *Brachiaria* cv Mulato II were selected because of their use in conventional push‐pull (using Silverleaf desmodium, *D. uncinatum*) and climate‐smart push‐pull (using Greenleaf desmodium, *D. intortum*). The two *Brachiaria* accessions, Piata and Xaraes, were recently introduced to farmers due to their superior performance over *Brachiaria* cv Mulato II. All plants used in this study were grown in pots (12 cm high, 13 cm diameter, with a single maize plant per pot) in screen houses on the field station and were 3–4 weeks old at the time of the experiment.

### 2.3 Fall armyworm colony

The pupae of FAW were obtained from a continuous colony reared in Mbita station for research purposes. Larvae were reared following the protocol described by Sokame et al. (2020).^22^ Pupae were placed in plastic containers until adult emergence. A cotton pad moistened with water was placed inside the container to maintain RH >80%. The insects were kept in a rearing room at 25 ± 3 °C, 75% RH and L12:D12 photoperiod. Emerged adult males and females were released in a wooden cage at the onset of the scotophase. The mating status was checked at hourly intervals until the end of the scotophase. Pairs of mating moths were collected in plastic jars. From these pairs, the gravid females were individually released in the mating cages the following night, with each containing one potted maize plant. Egg deposition was checked the following day. Neonates emerging from FAW egg batches were used for the experiments.

### 2.4 Larval behaviour and feeding

#### 2.4.1 *Larval orientation and settlement*

Larval orientation and settlement were assessed using a protocol described by Cheruiyot et al. (2018).^23^ Host plant preferences of FAW neonates for different companion plants were assessed in a two‐choice test. Experiments were conducted inside 15‐cm diameter Petri dishes lined with moist filter paper discs. Combinations of two 4‐cm leaflets (equivalent to a whole *Desmodium intortum* leaflet) of plant species were placed opposite one another, with their adaxial sides facing up. The following combinations were tested: maize *vs* desmodium, maize *vs Brachiaria* cv Mulato II, Maize *vs* bean, desmodium *vs Brachiaria* cv Mulato II, desmodium *vs* bean, and *Brachiaria* cv Mulato II *vs* bean. Ten FAW neonates were released at the centre of each Petri dish. The larvae were then allowed to settle on their preferred leaflet. A filter paper disc was placed inside the lid before closing, and then the lid was sealed with Parafilm to prevent the larvae from escaping. The Petri dishes were then placed in the dark by covering them with a black cloth. The number of larvae on each leaflet was recorded at 1 and 24 h after release to determine the larval orientation and the establishment, respectively. The leaflets with the highest and lowest numbers of larvae represented the most and least preferred plants for larval orientation and settling, respectively. This experiment was replicated 10 times.

#### 2.4.2 *Larval arrest and dispersal*

The leaflets of four plants, maize, bean, desmodium and *Brachiaria* cv Mulato II, were placed individually, with their adaxial side facing upwards, in the centre of a 9‐cm Petri dish lined with moist filter paper. Ten first instars of FAW were then introduced on top of each excised leaflet. A filter paper was lined up inside the Petri dish before sealing it with Parafilm to prevent the larvae from escaping. The Petri dishes were covered with a black cloth to keep them in the dark. The arrestment and dispersal behaviours were evaluated by counting the number of larvae that remained on the leaflet at 1 and 24 h after release. The leaflet with the highest and lowest numbers of larvae represented the most and least preferred plants for arrestment, respectively. The experiment was replicated 10 times.

#### 2.4.3 *Consumed leaf area*

This study was conducted to assess the feeding of FAW larvae on leaves of the four experimental plants used in intercropping: maize, bean, desmodium and *Brachiaria* cv Mulato II. Leaflets were placed in a 6‐cm diameter Petri dish lined with wet filter paper to limit desiccation. Each leaflet was placed in a different Petri dish. Ten newly hatched and unfed FAW larvae were placed on each leaflet. The Petri dishes were covered and sealed with Parafilm to prevent larvae from escaping and kept in the darkroom. The leaflet area (cm^2^) consumed by the larvae was calculated using the iOS LeafByte application.^24^ The most consumed leaves represented the most preferred plants for feeding. This experiment was replicated 10 times.

#### 2.4.4 *Larval survival and development*

The experiment was conducted for the duration of FAW larval stage. Screwed‐top transparent plastic jars (1 L) were filled with plant sections of maize, bean, *D. intortum* and *Brachiaria* cv Mulato II, sufficient to ensure larval feeding for 3 days. Plant sections consisted of young and tender leaves, as well as stem sections of 3–4‐week‐old plants. Moist paper towels were placed at the bottom of the jars to prevent desiccation. Twenty FAW neonates were released inside each jar. After placing the neonates, the lid of the jar was tightly closed with another paper towel to tighten the seal and prevent the larvae from escaping. The larvae were allowed to feed for 3 days, after which the plant sections were delicately removed from the jars to inspect the larvae.

Larvae were counted and weighed, and then returned to each jar with fresh plant material. This was repeated every 3 days until the pupation stage. Due to low weights of individual larvae, all living larvae in the same jar were weighed together, and the total weight (g) was divided by the number of larvae to obtain the mean weight, which was then used as an indicator of larval development. The percentage of survival was calculated for each jar on each survey date. The final percentage of survival was determined using the following formula:

$$\%\text{survival}=\left(\frac{\left(\;\text{number of larvae reaching pupation}\;\right)}{\text{number of larvae}\;\mathrm{at}\;\text{the beginning of the experiment}}\right)\times 100$$

Each treatment was replicated five times, and the experiment was repeated twice in August and September, respectively.

#### 2.4.5 *Oviposition preference*

The oviposition preference analysis was conducted using the protocol described by Khan et al.^21^ Two‐choice tests were carried out in oviposition cages (50 × 50 × 77 cm) covered with fine wire mesh netting. A piece of wet, damp cotton soaked in water was placed at the bottom of the cage as a water supply for the moths. Two potted 3–4‐week‐old plants of each plant species were placed in opposite corners of the cage. Five gravid naïve FAW moths were introduced in each cage half an hour before sunset under natural conditions. The moths were allowed to oviposit throughout the night. On the next day (16 h after release), each plant was examined and all egg masses were recovered. Four plant combinations were tested: maize *vs B. brizantha* cv Mulato II, *B. brizantha* cv Xaraes, *B. brizantha* cv Piata and *P. purpureum*. Each combination was repeated five times. The numbers of egg masses were recorded for each plant, and the number of eggs for each recovered mass was counted using a binocular magnifying glass. The plant with the highest number of eggs represented the most preferred host for oviposition.

### 2.5 Data analysis

Larval orientation and settlement experiments data were analysed using a generalised linear model assuming the binomial distribution error and logit link. However, due to over dispersion, quasi binomial model with logit link was used to explore the significance of differences in FAW preference of host plants. The total number of eggs deposited on host plants was analysed using a generalised linear model assuming the Poisson distribution and log link. However, due to overdispersion in the egg counts, we used the quasi‐Poisson model, which allows for estimation of dispersion parameter. The quasi‐Poisson model was used because the negative binomial model could not converge. ANOVA was used to determine the differences between the treatments, the host plants in larval arrest and dispersal, and for the consumed leaflet area experiments. The square root transformation was applied to comply with the conditions of normality and homogeneity of variance. Treatment means were separated using Tukey's honestly significant difference (HSD) procedure.

For the larval survival and development experiment, larval weight was analysed using the Kruskal–Wallis nonparametric test and multiple comparisons were carried out within the same trial. The test was performed once per survey date. Considering the high variability on weight between larvae and the level of cannibalism, the median was calculated instead of means. ANOVA was performed for the final percentage of survival, after a BLISS transformation. Pair‐wise differences between treatments were performed using the Tukey's HSD procedure and the level of significance was 5%. The analyses were implemented in R version 3.5.3.^25^

## 3 RESULTS

### 3.1 Larval orientation and settlement

**Figure 1:**
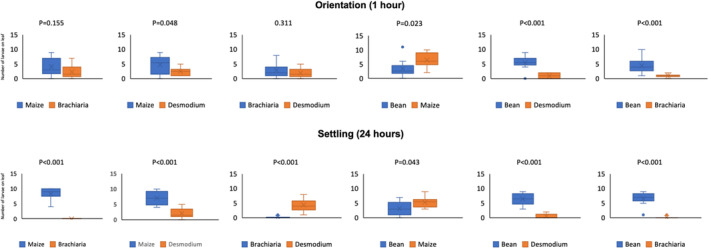
Median number of neonate larvae of *S. frugiperda* for orientation and settlement on leaflets of test plants 1 and 24 h after release, respectively. Numbers on the bar chart represents the *P* value following a generalised linear model with binomial distribution error and logit link.

One hour after the release of the neonates in the Petri dishes containing two choices of plant (tissue), the number of FAW larvae on maize was significantly higer than on desmodium and bean. The number of FAW larvae on bean was significantly higher than on desmodium and *B. brizantha* cv Mulato II (Fig. 1). There were no significant differences between the numbers of larvae on *B. brizantha* cv Mulato II and desmodium and between maize and *B. brizantha* cv Mulato II.

After 24 h, the differences were significant for all test plants (Fig. 1). FAW larvae preferred largely maize to desmodium and *B. brizantha* cv Mulato II, and the number of larvae orientating on maize was significantly higher than on bean. Bean came second in the larvae preferences, with *B. brizantha* cv Mulato II being the least preferred of all test plants. After 24 h, no larvae had chosen to stay on *B. brizantha* cv Mulato II for any of the test plant combinations (Fig. 1).

### 3.2 Larval arrest and dispersal

**Figure 2:**
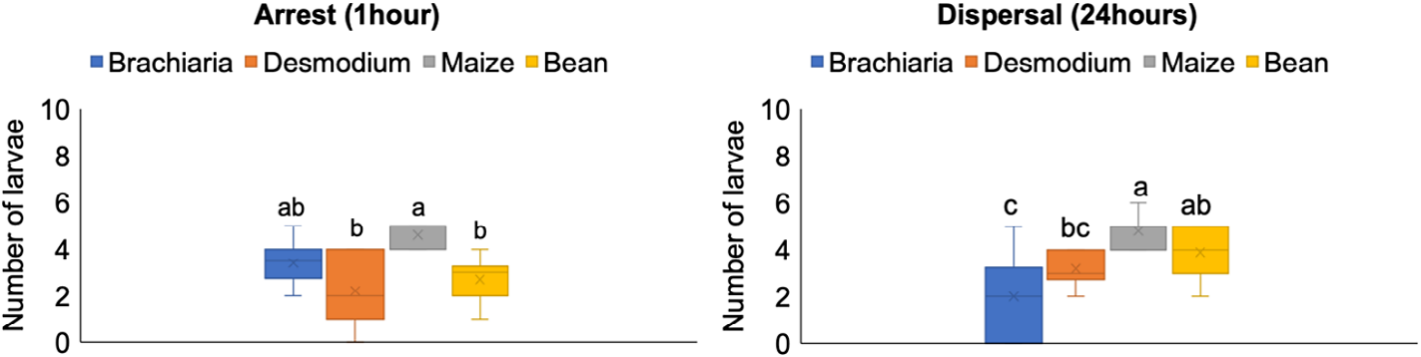
Mean (+SE) number of FAW larvae for arrest and dispersal on leaflets of test plants 1 and 24 h after release, respectively. (Means marked by different letters are significantly different by Tukey's studentised range test (*P* value < 0.05)).

One hour after release, the mean number of FAW larvae was significantly higher on maize than on bean and desmodium (Fig. 2). The mean numbers of larvae were not significantly different between maize and *B. brizantha* cv Mulato II. Likewise, the mean numbers of larvae were not significantly different between desmodium, bean and *B. brizantha* cv Mulato II.

After 24 h, the difference between maize and bean was no longer significant, but the mean number of larvae was significantly higher on maize than on desmodium or *B. brizantha* cv Mulato II (Fig. 2). The differences between desmodium and bean, and between desmodium and *B. brizantha* cv Mulato II were not significant. Maize has the highest mean number of larvae and *B. brizantha* cv Mulato II the lowest, following the same trend as the experiment on orientation and settling (Fig. 1).

### 3.3 Leaf area consumed

**Figure 3:**
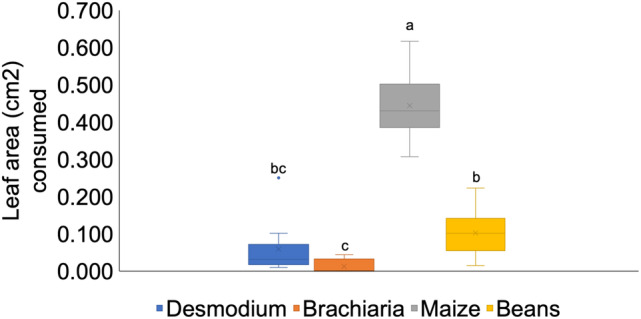
Mean (+SE) leaflet area (cm^2^) consumed by FAW larvae on leaflets of test plants 24 h after release (*n* = 10). (The means marked by different letters are significantly different by KrusMC multiple comparisons (*P* value < 0.05)).

The mean leaf area consumed by the larvae was significantly different between all test plants (*F*_3.39_ = 84.14, *P* < 0.0001), with maize being the most consumed plant, followed by bean, desmodium and finally *B. brizantha* cv Mulato II. There were no significant differences between desmodium and bean and between desmodium and *B. brizantha* cv Mulato II (Fig. 3).

### 3.4 Larval survival and development

**Figure 4:**
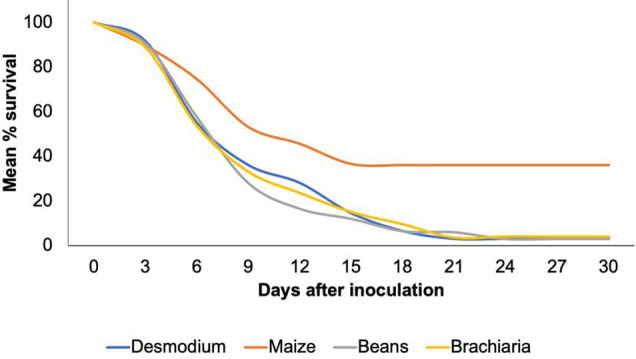
Mean final percentage of survival of FAW larvae fed with different test plants throughout all larval stages.

The mean percentage of survival to the pupation stage was significantly higher on maize (36%) than on all other test plants (*F*_3.39_ = 52.10, *P* < 0.0001) (Fig. 4). The percentages of survival were similar between bean, *B. brizantha* cv Mulato II and desmodium, ranging from 2.5% to 6%. FAW larvae were the heaviest on maize, although the differences with bean were not always significant (Table 1). The difference between maize and desmodium was significant throughout, both in August and in September, with the larvae being systematically heavier on maize than on desmodium. The difference between bean and Brachiaria was not significant at any time, both in August and in September. Bean, desmodium and *B. brizantha* cv Mulato II did not rank the same at all times in terms of mean larval weight. In both experiments, maize was the first test plant for which all larvae had either died or reached the pupation stage, with brachiaria being the last.

**Table 1:** Median weight of FAW larvae fed with test plants

Plant	Experiment 1		Experiment 2	
	Median	*Z* value	Median	*Z* value
6 days after release				
Maize	0.0510 ± 0.004a	3.27	0.0530 ± 0.0029a	3.27
Bean	0.0129 ± 0.003ab	0.83	0.0061 ± 0.0009ab	−0.48
Desmodium	0.0015 ± 0.003b	−2.57	0.0045 ± 0.0042b	−1.66
Brachiaria	0.0037 ± 0.000b	−1.53	0.0047 ± 0.0004b	−1.13
9 days after release				
Maize	0.3016 ± 0.0140a	3.27	0.3390 ± 0.0179a	3.27
Bean	0.0590 ± 0.0137ab	0.48	0.0200 ± 0.0270b	−1.18
Desmodium	0.0080 ± 0.0015b	−3.27	0.0176 ± 0.0095b	−1.61
Brachiaria	0.0328 ± 0.0299ab	−0.48	0.0273 ± 0.0047ab	0.48
12 days after release				
Maize	0.4400 ± 0.0471a	3.27	0.2529 ± 0.2546a	2.78
Bean	0.1201 ± 0.0165ab	0.26	0.0999 ± 0.0948ab	0.50
Desmodium	0.0307 ± 0.0032b	−3.27	0.0538 ± 0.0346b	−2.22
Brachiaria	0.0857 ± 0.0052ab	−0.26	0.0852 ± 0.0182ab	−1.02
15 days after release				
Maize	0.5140 ± 0.1447a	3.27	—	–
Bean	0.2230 ± 0.0106ac	1.09	—	–
Desmodium	0.0978 ± 0.0216b	−3.27	—	–
Brachiaria	0.1790 ± 0.0062bc	−1.09	—	–

Medians marked by different letters are significantly different by KrusMC multiple comparisons (*P* value < 0.05) (*n* = 5).

### 3.5 Oviposition preference

**Figure 5:**
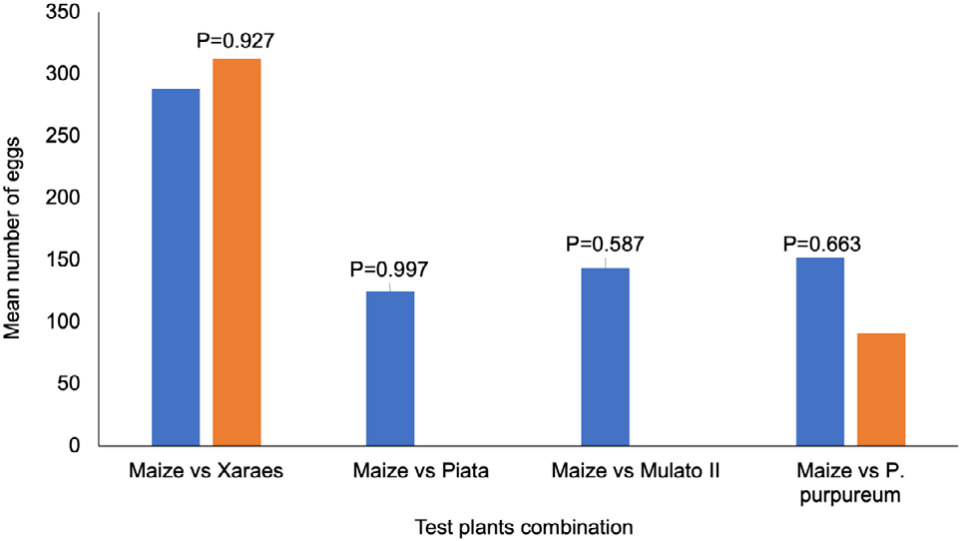
Oviposition preferences of FAW moths between maize and different trap plants (*n* = 5). (Numbers on bar chart represent the *P* values following a generalised linear model with binomial distribution error and logit link).

Although the total number of eggs deposited by FAW moths was higher on *B. brizantha* cv Xaraes, no significant differences were found between the tested host plants (*P* > 0.05) (Fig. 5).

## 4 DISCUSSION

Host plants are key components of the ecological niche of herbivorous insects, through their roles as a food resource, overwintering refuge, oviposition sites and, in some cases, mating sites.^26^ Insects detect and select their host plants through complex biological processes that have evolved over time.^27^ In the absence of the most preferred host, alternative host plants (usually belonging to the same family as the primary host plant) can ensure the continuity of the pest. Hence, the choice of companion plants becomes a crucial element in agroecological pest management of FAW.^28^, ^29^ Although FAW can feed on more than 300 host plants,^13^, ^20^ it prefers maize by far. The study showed that bean remained FAW's second most preferred plant, although the percentage of mortality of larvae feeding on it was as high as that in desmodium and Brachiaria. Larvae did not gain as much weight as on maize.

**Figure 6:**
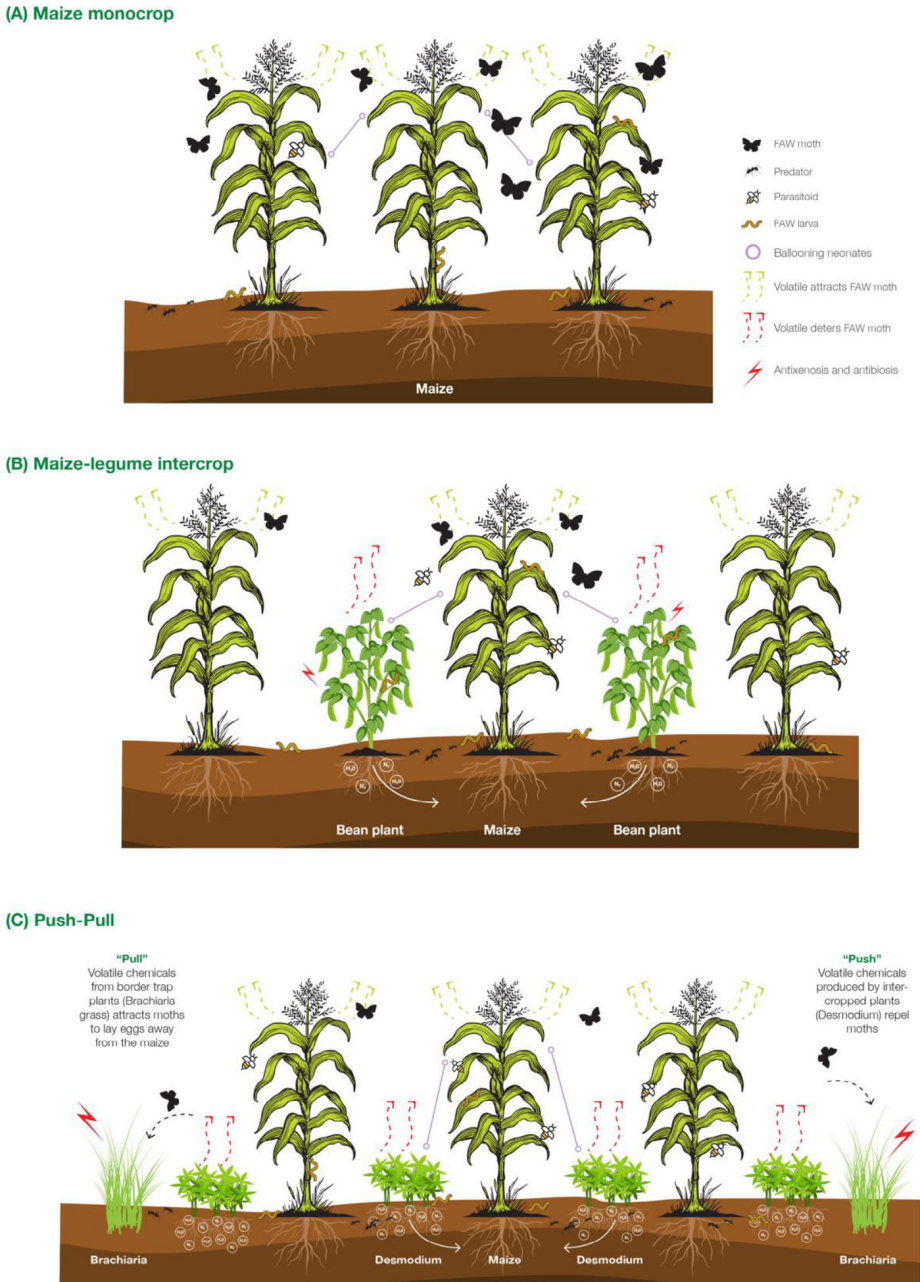
FAW control mechanisms under maize‐legume intercropping and push‐pull compared to monocropping.

Plant defenses against herbivores can be mechanical or chemical, and these defense mechanisms are the result of many chemical and morphological adaptations.^30^, ^31^, ^32^ Desmodium and Brachiaria had a higher deterrent effect on FAW larvae and this resulted also in high mortality rates, compared with maize. The findings described in this study provide a better understanding of FAW control under monocrop, bean intercropping and push‐pull cropping systems (Fig. 6). Compared with monocropping (Fig. 6(a)), intercropping systems are disrupting mechanisms that interfere with insect‐host–plant relationships (Fig. 6(b)). Within a maize monocrop plot, FAW neonates spread by crawling or ballooning, with a wider dispersal and plant damage potential.^22^ In the push‐pull system (Fig. 6(b)), the legume intercrop, desmodium is deterrent to stemborer moths as it produces repellent volatile chemicals such as (*E*)‐β‐ocimene and (*E*)‐4,8‐dimethyl‐1,3,7‐nonatriene, which are responsible for repellency to stemborers.^33^ Desmodium causes an antixenosis effect on FAW larvae and may act as a physical barrier to prevent larval movement in the field, as some species of desmodium, e.g. *Desmodium uncinatum*, are particularly hairy or sticky and can trap larvae that disperse among plants and rows.^14^, ^20^ Similarly, antixenosis and antibiosis effects have been reported in bean cultivars on FAW larvae.^34^ Moreover, bean plants may also act as physical barriers as trichome‐based plant defense mechanisms have also been observed.^35^

Larvae reared on maize were healthier in size and weight compared with others. The deterrent effect of the companion plants is characterized by an extension on the larval stage resulting in negative effects on FAW developmental stages, high mortality and decreased body weights. Intercropping with bean or desmodium reduces FAW larval fitness and therefore its ability to spread in the maize fields.^14^

The results of the leaflet area consumed experiment showed that brachiaria, although belonging to the same family as maize, acts as an effective dead‐end trap crop for FAW larvae, contributing to suppressing pest populations in the fields. Similar results were also reported on *C. partellus*; first instars did not consume leaves of brachiaria plants but consumed those of maize, which also suffered more stem damage than Brachiaria plants did.^23^

Although bean was not the most preferred crop, FAW larvae can survive on it, as compared with desmodium or brachiaria. Our results therefore justify the lower efficacy of edible legume intercropping (30% and 40% reduction in FAW infestation), compared with push‐pull (82% FAW reduction), in the reduction of FAW infestation.^15^, ^16^, ^36^ Our results also justify the lower incidence of FAW in regions where cereal‐legume intercropping is part of cultural practice compared to regions where monocropping is more frequent.

The study on oviposition preference showed that FAW moth prefers to lay eggs on maize compared to *B. brizantha* cv Piata, Mulato II or Napier grass. Only *B. brizantha* cv Xaraes showed equal oviposition preference. These findings confirm previous reports on FAW oviposition preferences on maize compared with other grasses.^37^, ^38^ The *Chilo partellus* moth prefers *B. brizantha* to maize for oviposition.^39^ The underlying mechanisms for higher preference for *B. brizantha* cv Xaraes are unknown. However, several complex factors underpin insect host preference; for example leaf texture plays a critical role in oviposition preference. Certain varieties of brachiaria are not preferred for oviposition due to the strong presence of trichomes on the leaf surface.^23^ In both FAW and *C. partellus*, brachiaria supports minimal feeding and survival of larvae. However, there is still need for the screening of other potential trap crops which are more attractive to FAW for oviposition than maize.

This study explains partially the mechanisms involved in the control of FAW under the push‐pull and legume intercropping systems. The repellent effect of Desmodium forces gravid moths to lay eggs on Brachiaria cv Mulato II and *Pennisetum purpureum*. Intercropping limits the potential of neonates to spread while at the same reducing the chance of oviposition by FAW moths. Our study demonstrates that FAW neonates cannot survive on brachiaria.

In the push‐pull system, the attractiveness of the trap crop is crucial. Our results show that *B. brizantha* cv *Xaraes* stands as a promising trap crop for the management of FAW in push‐pull; however, further studies with a broader range of plants would be necessary.

One advantage with leguminous intercrops such as Desmodium and bean is that they improve soil fertility management through nitrogen fixation, which also contributes to overall plant health. Further studies on plant‐induced responses on FAW natural enemies might provide additional information. Crop diversification has been shown to reduce pest infestation and oviposition on crop plants, either by emitting volatiles that directly repel gravid females or by providing olfactory camouflage.^14^ Natural enemies respond to plant volatiles that are released when the plant is damaged by a herbivore. Studies have also demonstrated that parasitoids could be recruited in anticipation of egg hatching due to the presence of an elicitor, which could be extracted from egg materials associated with attachment to leaves. Genome‐wide association studies (GWAS) on maize cultivars have identified markers associated with plant defence.^40^ Such findings suggest selective planting of companion plants to protect maize against FAW.
